# Intravascular Myopericytoma of the Foot: A Diagnostic Challenge

**DOI:** 10.7759/cureus.85110

**Published:** 2025-05-30

**Authors:** Taro Fujimaki, Tomonori Kawasaki, Masanori Wako, Kojiro Onohara, Jiro Ichikawa

**Affiliations:** 1 Department of Orthopedic Surgery, University of Yamanashi, Chuo, JPN; 2 Department of Pathology, Saitama Medical University International Medical Center, Hidaka, JPN; 3 Department of Radiology, University of Yamanashi, Chuo, JPN

**Keywords:** differential diagnosis, histopathology, imaging, intravascular, myopericytoma

## Abstract

Myopericytoma is a rare pericytic tumor, typically occurring in the skin and subcutaneous tissue, though cases in internal organs and bones have been reported. Clinically, it presents as a painless, slow-growing mass, making differential diagnosis challenging. Imaging alone is often insufficient due to overlapping characteristics with angioleiomyoma, glomus tumors, and solitary fibrous tumors (SFT). Histopathological examination is essential for accurate diagnosis. Here, we report a rare case of intravascular myopericytoma of the foot. MRI findings suggested multiple differential diagnoses, but histopathology confirmed the tumor. Immunohistochemistry supported the diagnosis. Marginal excision was performed, and no recurrence was observed after three years. Given the overlapping imaging characteristics of pericytic tumors, histopathological analysis is crucial for distinguishing myopericytoma from similar entities and ensuring proper treatment and management. This case highlights the importance of comprehensive evaluation when diagnosing pericytic tumors in distal extremities.

## Introduction

Myopericytoma was first reported by Granter et al. in 1998 in seven cases [1]. It is currently classified as a pericytic tumor [2]. Myopericytomas are rare tumors that primarily develop in the skin and subcutaneous tissues, although occasional cases of internal organ or bone involvement have been documented [2,3]. Clinically, it is characterized by painless and slow growth. Although imaging is useful to confirm the presence of a tumor, its distinctive features for differential diagnosis remain unclear [4,5]. In cases involving the distal extremities, the small tumor size makes it difficult to determine, often challenging an accurate diagnosis based solely on imaging [6]. Histopathological examination is crucial for a correct diagnosis, but differentiating myopericytoma from other pericytic tumors, including angioleiomyoma and glomus tumors, can be difficult because of overlapping histological features [7]. Additionally, variants such as intravascular [2] and malignant myopericytomas [8] have been reported, further complicating the pathological diagnosis. In most cases, marginal resection results in a good prognosis [2]. Here, we report an extremely rare case of intravascular myopericytoma of the foot, where the differential diagnosis was complex and there were challenges in obtaining the imaging evidence.

## Case presentation

**Figure 1:**
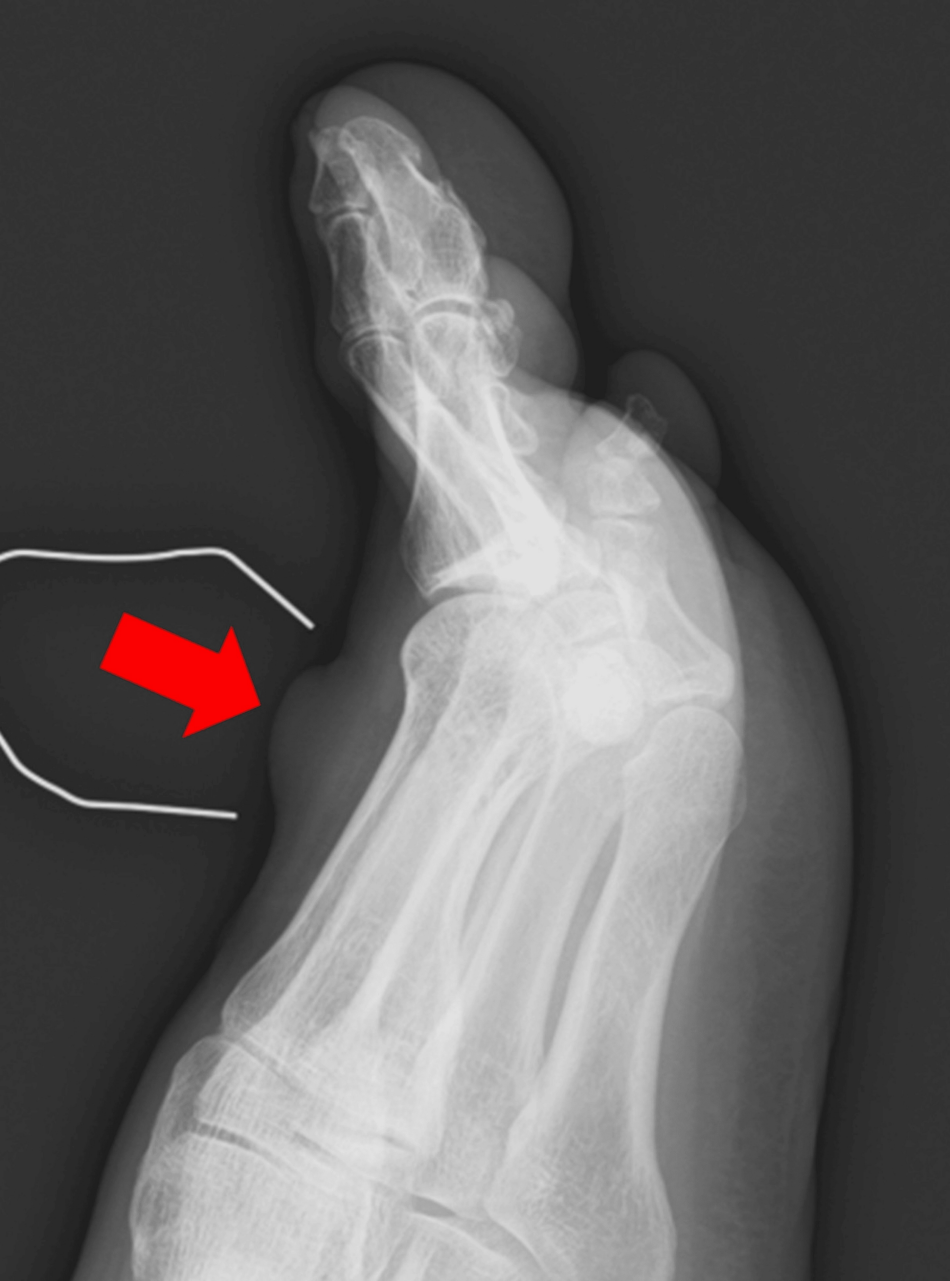
Plain radiography No soft tissue calcifications or abnormalities in the surrounding bone were observed. The white lines represent the metal marker used during the X-ray examination

The patient was a 71-year-old male who had a mass on the dorsum of his left foot for eight years, showing a gradual increase in size, prompting him to visit our hospital. The mass was located on the medial side of the dorsum of the left foot, with a dark reddish skin coloration. There was slight tenderness on palpation but no spontaneous or cold-induced pain. Additionally, Tinel's sign was not observed. The patient could walk and had no prior medical history, and all blood test results were within the normal range. Plain radiographs (Figure 1, red arrow) revealed no soft tissue calcifications or bone abnormalities.

**Figure 2:**
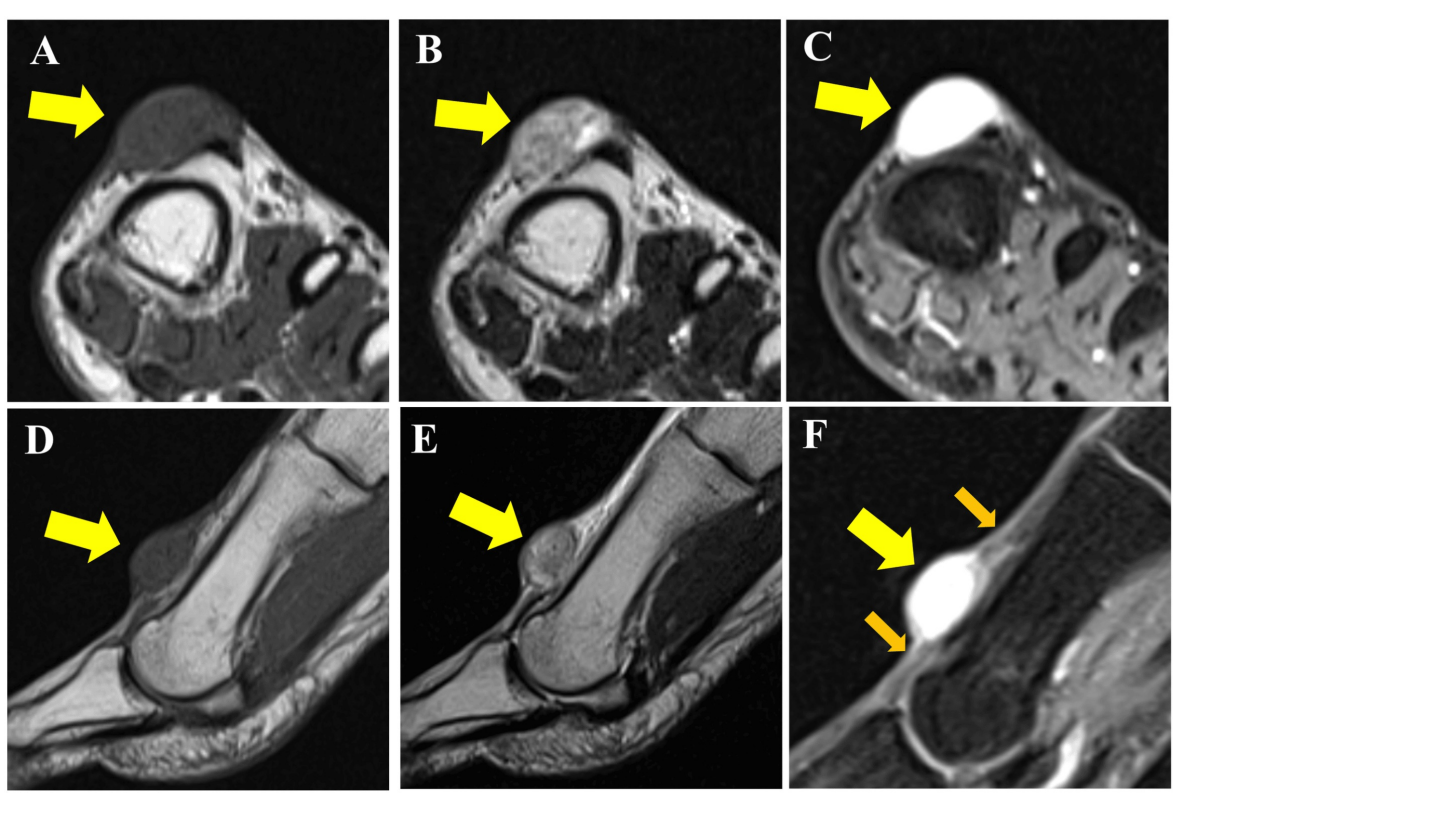
MRI findings Axial (A-C) and sagittal (D-F) views. There is a well-defined subcutaneous mass with a clear boundary and no invasion into bone. Low signal intensity on T1-weighted imaging (A, D, yellow arrow), heterogeneous high signal intensity on T2-weighted imaging (B, E, yellow arrow), and avid enhancement on contrast-enhanced T1-weighted imaging (C, F, yellow arrow). The proximity between a blood vessel and the tumor (F, orange arrows)

MRI revealed that the tumor was located in subcutaneous tissue, showing a low signal intensity on T1-weighted imaging (Figures 2A, 2E), a heterogenous high signal intensity on T2-weighted imaging (Figures 2B, 2E), and an avid enhancement on contrast-enhanced T1-weighted imaging (Figures 2C, 2F). Based on the MRI findings, differential diagnoses included angioleiomyomas, solitary fibrous tumors (SFT), and schwannomas.

**Figure 3:**
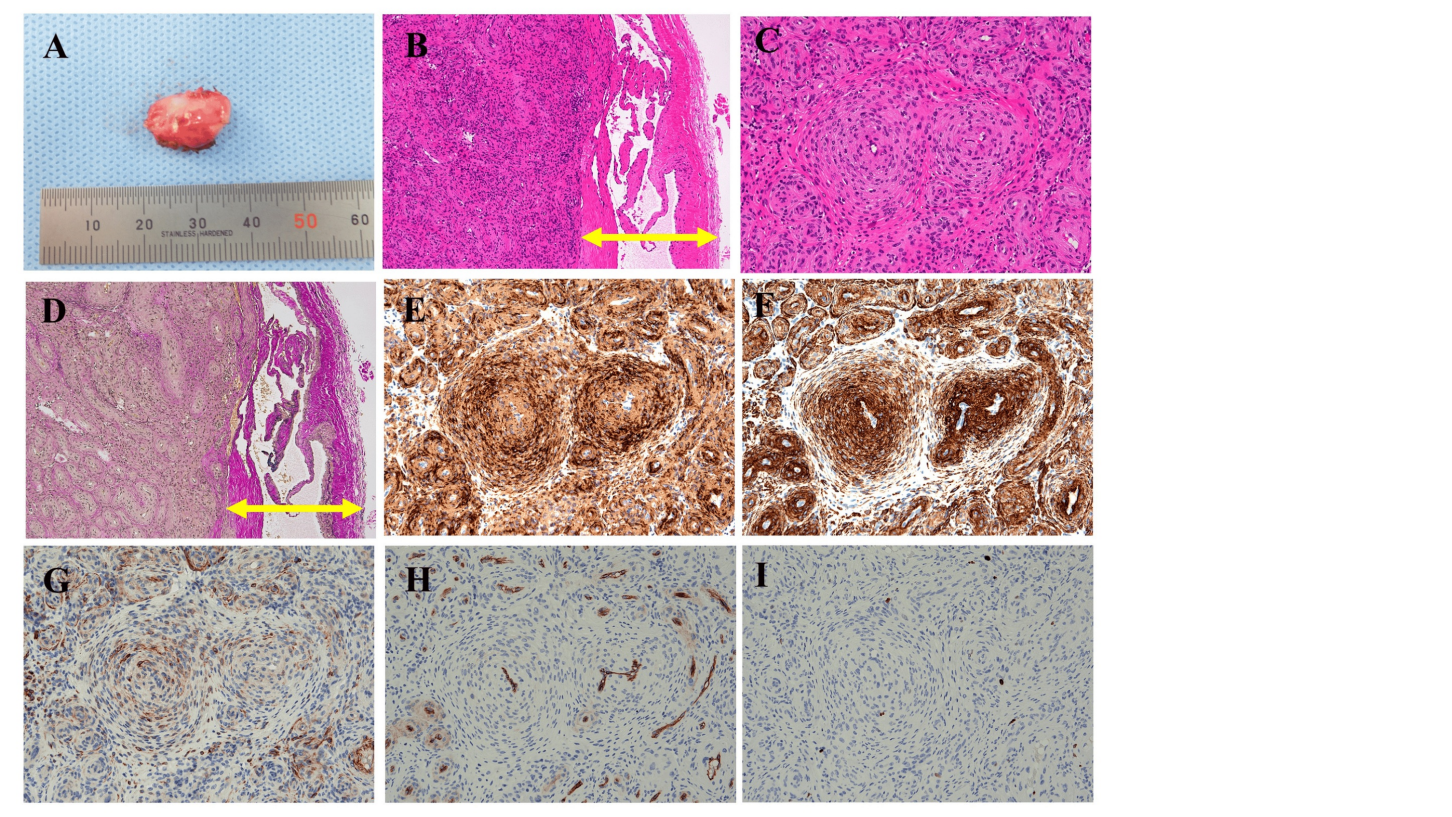
Macroscopic and microscopic findings Macroscopic and microscopic findings. (A) Macroscopic findings of the tumor showed a grayish-white appearance with a size of 20 × 10 × 10 mm. Histopathological findings indicated an association with blood vessels (B and D, yellow double arrow indicates vascular wall), with numerous blood vessels present within the tumor, surrounded by the spindle-shaped cells proliferating in a layered pattern (C). Elastica van Gieson staining (D). Immunohistochemistry (IHC) results: α-SMA (E), h-caldesmon (F), desmin (G), CD34 (H), and MIB-1 (I) (magnifications: B, D: ×100; C, E-I: ×200)

Owing to the small tumor size, we decided to perform an excisional biopsy and explained to the patient that an additional resection would be performed if a malignancy was detected. A marginal resection was performed. Macroscopically, the tumor was grayish-white and measured 20 × 10 × 10 mm (Figure 3A). Histopathological examination revealed numerous blood vessels surrounded by spindle-shaped cells proliferating in a layered, concentric pattern (Figures 3B-3D). Immunohistochemistry (IHC) showed positivity for α-SMA (Figure 3E) and h-caldesmon (Figure 3F), focal positivity for desmin (Figure 3G), and negativity for CD34 (Figure 3H). The Ki67 (MIB-1) labeling index was 1% (Figure 3I).

The tumor was located within a large blood vessel and diagnosed as an intravascular myopericytoma. Three years after surgery, there was no obvious recurrence.

## Discussion

Myopericytomas are perivascular myoid neoplasms classified as pericytic tumors and include angioleiomyomas, glomus tumors, and myofibromas [2]. Myopericytomas typically arise in skin or subcutaneous tissues. Regarding its site of occurrence, it is most commonly found in the extremities, followed by the head and neck, and the oral cavity, with a predilection for the lower limbs. Rare cases have been reported in internal organs, intracranial regions, and bones [2,3]. Clinically, this condition is characterized by slow and painless growth [2]. Although it can occur at any age, it is most prevalent in individuals aged 40-50 years and tends to affect the male population more frequently. The average tumor size is approximately 2 cm; however, tumors exceeding 10 cm have also been reported [2,9]. In this case, the site of occurrence, clinical features, sex, and tumor size were consistent with previously reported findings. A characteristic symptom difference between myopericytoma, angioleiomyoma, and glomus tumors is the presence of pain, which is significantly higher in angioleiomyoma [10] and glomus tumors [11]. From another perspective, intravascular myopericytomas are reported to have a higher frequency of pain compared to that of conventional myopericytoma [12,13]; however, there was no pain observed in our case. Although the pathogenesis of myopericytoma remains unclear, in cases of intravascular myopericytoma, venous stasis and minor trauma have been reported as contributing factors [12]. However, neither was observed in this case. Another characteristic of myopericytoma is slow growth; in this case, the tumor has been present for eight years. Typically, such an indolent course may suggest a benign tumor; however, cases of synovial sarcoma with slow growth and minimal pain have also been reported [14]. Synovial sarcomas occurring in the hands and feet have been reported to have a median tumor size of 3.3 cm, with some cases showing a prolonged course of up to 30 years [14]. Therefore, relying solely on the clinical course and findings to classify a tumor as benign can sometimes be challenging, and sarcoma should always be considered as a differential diagnosis.

Radiographs generally show no significant abnormalities [5]. In typical cases of myopericytoma, MRI reveals low signal intensity on T1-weighted images, high signal intensity on T2-weighted and fat-suppressed T2-weighted images, and marked contrast enhancement. These findings are similar to those of angioleiomyomas and glomus tumors [4,5,10,11]. MRI findings of the SFT, which was one of the differential diagnoses in our case, often show intermediate to high signal intensity on T2-weighted and short tau inversion recovery (STIR) imaging with avid contrast enhancement as a characteristic feature [15]. Interestingly, the MRI findings in our case of indolent synovial sarcoma were similar to those of myopericytomas [14]. Currently, the characteristic MRI findings of myopericytoma include (1) absence of peritumoral edema, (2) presence of blood vessels within the tumor, (3) continuity or proximity to blood vessels, and (4) nodules within the tumor showing low signal intensity on T2-weighted imaging. However, the sensitivity and specificity of these findings remain unclear and require further analysis [4,5]. Interestingly, SFT often share imaging characteristics with myopericytomas, such as the presence of abundant microvasculature within the tumor and the absence of surrounding edema. However, SFT tend to originate in deeper tissues and are generally larger, which serves as a key distinguishing factor [15]. Additionally, tumors in the peripheral extremities are often small, making it difficult to identify the characteristic imaging features of myopericytomas. Previous cases of intravascular myopericytoma arising in the foot were similar to our case [6]. Given these considerations, it appears that MRI alone is insufficient for accurately differentiating not only pericytic tumors but also benign and malignant lesions.

Histopathological examinations are essential for diagnosing myopericytoma. Histologically, myopericytomas consist of oval to spindle-shaped myoid cells, with tumor proliferation forming multilayered concentric structures around blood vessels. Intravascular myopericytomas showed similar findings [2,13]. However, the morphological spectrum of myopericytoma is broad, with angioleiomyoma-, myofibroma-, and glomus-like variants reported [9]. Given the inherent overlap among pericytic tumors, their distinction is often challenging [7]. Recent reports have identified PDGFRB and NOTCH3 mutations as characteristics of myopericytoma; however, these mutations have also been observed in angioleiomyoma and glomus tumors, suggesting that they are not useful for differential diagnosis [7]. In IHC of myopericytoma, α-SMA and h-caldesmon are positive, while desmin and CD34 show focal positivity. In contrast, they are negative for S-100 and epithelial markers [2,9]. Although extremely rare, malignant myopericytomas have been reported [8], and these tumors grow rapidly and exhibit high degrees of malignancy. Histologically, the key differentiating features between benign myopericytomas and malignant variants include pleomorphism, dense cellularity, and increased mitotic activity [8]. In malignant myopericytoma, epithelial membrane antigen positivity has been observed, although the reported positivity rate is only 25%, raising questions regarding its diagnostic utility [8]. Further studies are required to determine the necessity of appropriate surgical margins, chemotherapy, and radiation therapy.

Primary treatment with marginal resection was sufficient [2]. Although rare, cases of recurrence have been reported, including those of intravascular and malignant myopericytomas [9]. In terms of intravascular myopericytoma, rather than true recurrence, these cases are considered to represent the continuous progression of the tumor [2]. Additionally, radiation therapy has been used in cases of incomplete bone resection, but its effectiveness remains uncertain [3]. Since there have been no reports of malignant transformation to date, additional resection should be sufficient, even in cases of recurrence.

## Conclusions

Herein, we present a case of intravascular myopericytoma in which differentiation based on imaging findings and symptoms was challenging, with no clinical findings that are often observed in intravascular myopericytoma. Distinguishing myopericytoma from other pericytic tumors, such as angioleiomyomas and glomus tumors, using imaging alone is impractical. Furthermore, differential diagnosis encompasses a wide range of conditions, including SFT, schwannomas, and malignant soft tissue tumors. Given the slow growth and small size of the tumor, it is crucial not to be misled by these factors and to ensure an accurate diagnosis through histopathological examination.
